# Interface Engineering for the Enhancement of Carrier Transport in Black Phosphorus Transistor with Ultra-Thin High-*k* Gate Dielectric

**DOI:** 10.1038/srep26609

**Published:** 2016-05-25

**Authors:** Zhi-Peng Ling, Jun-Tao Zhu, Xinke Liu, Kah-Wee Ang

**Affiliations:** 1 Department of Electrical and Computer Engineering, National University of Singapore, 4 Engineering Drive 3, Singapore 117583; 2 College of Materials Science and Engineering, Shenzhen Engineering Laboratory for Advanced Technology of Ceramics, Nanshan District Key Lab for Biopolymer and Safety Evaluation, Shenzhen University, 3688 Nanhai Ave, Shenzhen 518060, People’s Republic of China

## Abstract

Black phosphorus (BP) is the most stable allotrope of phosphorus which exhibits strong in-plane anisotropic charge transport. Discovering its interface properties between BP and high-*k* gate dielectric is fundamentally important for enhancing the carrier mobility and electrostatics control. Here, we investigate the impact of interface engineering on the transport properties of BP transistors with an ultra-thin hafnium-dioxide (HfO_2_) gate dielectric of ~3.4 nm. A high hole mobility of ~536 cm^2^V^−1^s^−1^ coupled with a near ideal subthreshold swing (*SS*) of ~66 mV/dec were simultaneously achieved at room temperature by improving the BP/HfO_2_ interface quality through thermal treatment. This is attributed to the passivation of phosphorus dangling bonds by hafnium (Hf) adatoms which produces a more chemically stable interface, as evidenced by the significant reduction in interface states density. Additionally, we found that an excessively high thermal treatment temperature (beyond 200 °C) could detrimentally modify the BP crystal structure, which results in channel resistance and mobility degradation due to charge-impurities scattering and lattice displacement. This study contributes to an insight for the development of high performance BP-based transistors through interface engineering.

The potential of atomically thin two-dimensional layered materials in meeting the performance requirements of international technology roadmap for semiconductors^1^ has spun off intense research efforts on graphene^2^,^3^, transition metal dichalcogenides (TMD)^4^, and most recently black phosphorus (BP) crystal^5^. In particular, BP is known to be a promising channel material for future nanoelectronics applications owing to its superior carrier transport and direct bandgap properties for all layer thicknesses. This ranges from ~0.3 eV in the bulk form^6^,^7^ and increases to ~2 eV when the BP thickness is reduced to monolayer form^8^. Excellent electron and hole mobilities reaching ~15,000 cm^2^V^−1^s^−1^ and ~50,000 cm^2^V^−1^s^−1^, respectively, have also been reported in bulk BP single crystal^7^. Although a handful of BP transistors have been previously demonstrated^9^,^10^,^11^,^12^,^13^,^14^,^15^,^16^,^17^, the device performance is still far from satisfactory. Furthermore, to be CMOS compatible, the integration of a high-temperature thermal anneal is typically required to improve device performance. Some initial studies on the role of temperature on BP film structure and device performance have been reported^18^,^19^,^20^,^21^,^22^. With increasing anneal temperatures, Liu *et al*.^19^ showed the decomposition of BP film at 400 °C and a temperature induced increase in lattice parameters as confirmed by *in situ* scanning/transmission electron microscopy measurements in the vacuum state. For practical applications, surface passivation of the BP channel is necessary to address photo-oxidation issue^23^. When BP is passivated with hexagonal boron nitride (h-BN), an insulating 2D material, the device shows a significantly higher temperature tolerance up to 500 °C^18^. The high temperature anneal can help to suppress the charge trap states, leading to low hysteresis, improved mobility and high on-off ratios (>10^5^). Although h-BN is a possible candidate for device integration, the relatively low dielectric constant (<4)^24^ severely limits its potential for highly scaled transistors. Thus, there is a demand for suitable high-*k* gate dielectrics to improve the device performance. One possible high-*k* dielectric candidate is hafnium-dioxide (HfO_2_) which has a dielectric constant of ~25^25^, which is six times higher than the conventional SiO_2_ dielectric. Although some preliminary BP transistors have been realized on HfO_2_ or Al_2_O_3_ gate dielectric, poor subthreshold swing performance of the order of 0.3~1.1 V/dec and carrier mobility below ~310 cm^2^V^−1^s^−1^ were reported. Moreover, there are limited reports on the application of thermal treatment to improve the BP/high-*k* interface quality for enabling further performance enhancement.

In this work, we report the realization of BP transistors with a near ideal subthreshold swing (~66 mV/dec) and enhanced hole mobility (~536 cm^2^V^−1^s^−1^) through the use of an ultra-thin hafnium-dioxide (HfO_2_) gate dielectric and interface engineering. We further investigate the influence of thermal treatment on the structural and electrical properties of the BP transistors. Detailed material studies are performed using X-ray photoelectron spectroscopy, Raman spectroscopy and energy-dispersive X-ray spectroscopy to analyze the structural integrity and interface properties between the BP and HfO_2_ gate dielectric. Comprehensive electrical performance metrics including subthreshold swing, hole mobility, transfer characteristics, and channel resistance are systematically analyzed.

## Results and Discussion

**Figure 1:**
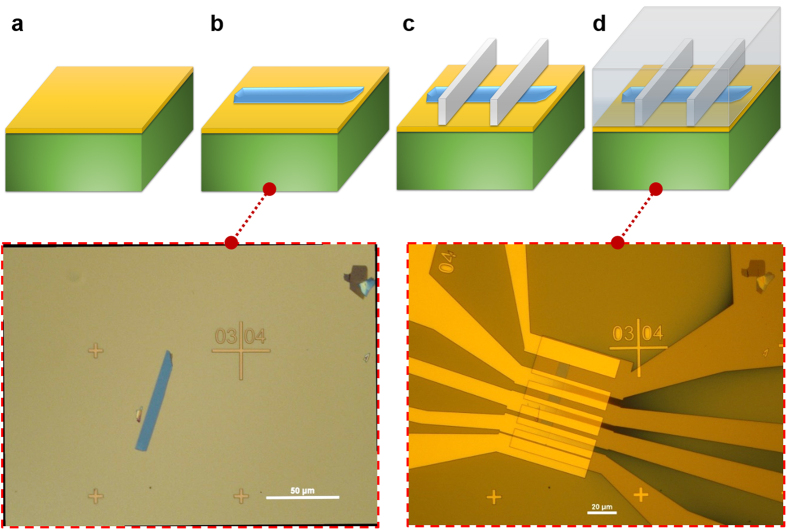
The fabrication flow for the black phosphorus transistor based on low temperature CMOS-compatible processes. (**a**) Using a degenerately doped Si substrate, an ultra-thin hafnium-dioxide gate dielectric of ~3.4 nm is deposited using atomic layer deposition (ALD) technique at a temperature of 250 °C. (**b**) BP nanosheet with a ~15 nm thickness is micromechanically exfoliated onto the high-*k* gate dielectric. (**c**) Metal electrodes utilizing nickel with a thickness of 120 nm are formed by a combination of electron beam lithography and electron-beam evaporation followed by the lift-off process. (**d**) An additional surface passivation by a 30 nm thick SiO_2_ layer is deposited to protect the as-fabricated devices from photo-oxidation in ambient conditions.

**Figure 2:**
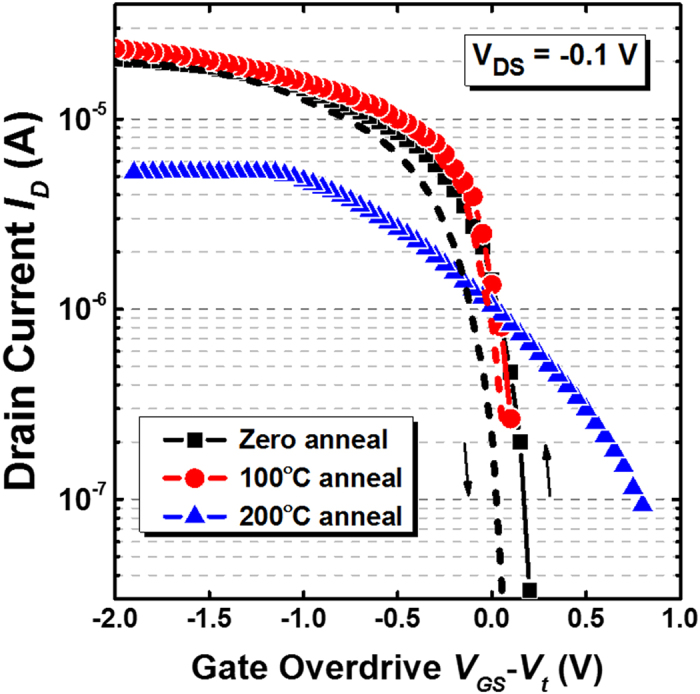
A comparison of the transfer characteristics of the black phosphorus transistors with and without interface annealing. With an excessively high thermal budget, the subthreshold swing of the BP transistor can be significantly degraded, which is attributed to the detrimental change of the BP film crystallinity. When subjected to a 100 °C thermal anneal, an off-state current of ~8.09 × 10^−7^ A was measured in the BP transistor. However, an improved hysteresis performance was estimated in devices which were annealed at 100 °C as compared to the un-annealed devices. This further confirms the achievement of lower *D*_*it*_ due to an optimum thermal annealing.

**Figure 3:**
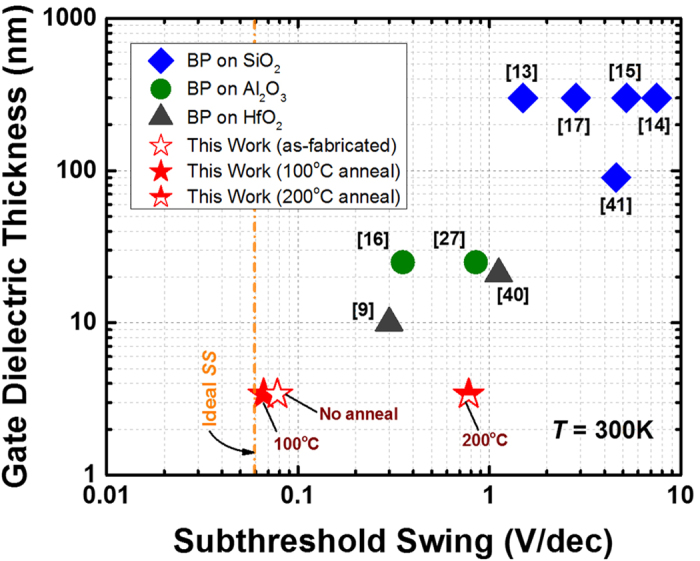
Figure-of-merit showing the relationship between gate dielectric thickness and the subthreshold swing (*SS*) performance of black phosphorus transistors. A significant *SS* improvement is observed by downscaling the thickness to ~3.4 nm coupled with an interface anneal process, showing a near ideal value of ~66 mV/dec. This is by far the lowest *SS* reported, which indicates an enhanced electrostatics control in the BP transistors.

**Figure 4:**
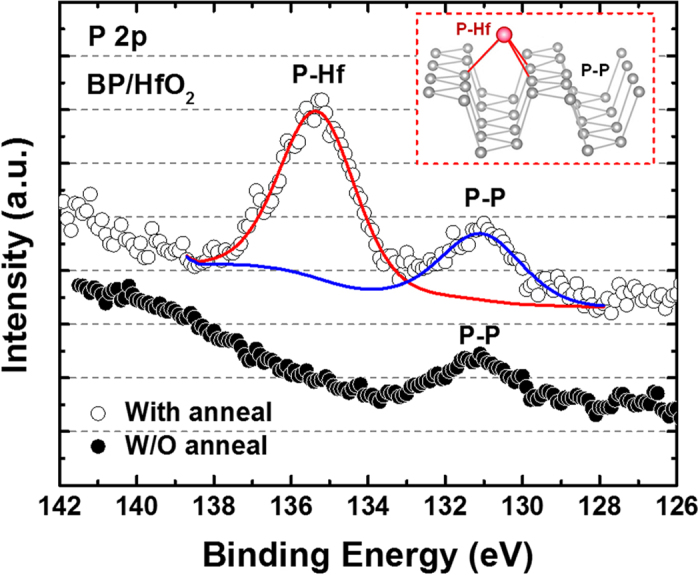
The P 2p core level of X-ray photoelectron spectra (XPS) for the black phosphorus nanosheet formed on HfO_2_ gate dielectric. A thermal treatment at 100 °C under nitrogen ambient improves the binding energy of P-Hf, which allows the achievement of better interface quality between BP and HfO_2_. The inset shows the formation of covalent bonds between P and Hf adatoms with a strong binding energy of −4.17 eV, as confirmed by the first-principles calculation within the density-functional theory (DFT) framework.

Figure 1 shows the low temperature fabrication flow used to realize the black phosphorus (BP) transistor with an ultra-thin high-*k* gate dielectric. The devices feature a back-gate configuration with a minimum gate length of ~3 μm. A CMOS-compatible metal such as nickel (Ni) was used to form the metal electrodes^26^. The as-fabricated transistor was passivated with a 30 nm silicon-dioxide (SiO_2_) layer deposited by e-beam evaporator at room temperature to protect the device from photo-oxidation in the ambient condition. A detailed description of the process is provided in the experimental method section. The devices realized in this work demonstrate a clear p-type behavior as depicted in Fig. 2, in which the room temperature transfer curves (*I*_*DS*_ - *V*_*G*_) are plotted at the same drain voltage (*V*_*DS*_) of −100 mV. In the as-fabricated state, the BP transistor shows a significantly improved subthreshold swing (*SS*) of ~76 mV/dec as compared to other BP transistors based on traditional SiO_2_ gate dielectric (*SS* > 1 V/dec)^9^,^10^,^11^,^12^,^13^,^14^,^15^,^16^,^17^ or Al_2_O_3_ gate dielectric (*SS* ~ 350–850 mV/dec)^15^,^27^, as benchmarked in Fig. 3. The improved *SS* performance implies the effectiveness of ultra-thin high-k gate dielectric in providing excellent gate control over the source-to-drain current, which enables BP transistor to achieve better electrostatics control. When subjected to an additional interface anneal for one minute under nitrogen (N_2_) ambient, the *SS* performance was shown to improve further and reaching a near ideal value of 66 mV/dec at room temperature (*T* = 300 K). Such a significant improvement is resulted from a better BP/HfO_2_ interface due to the passivation of phosphorus dangling bonds by hafnium (Hf) adatoms as promoted by the thermal treatment. This can be evidenced from the strong P-Hf binding energy at ~135.6 eV over the P-P binding energy at ~131.1 eV, as confirmed by the P 2p core level obtained from X-ray photoelectron spectroscopy (XPS) measurement in Fig. 4. The binding energies of all spectra are referenced to C1s which is set to 285 eV. The appearance of P-P peak or the single spin-orbit split doublet at a binding energy of ~131 eV indicates the intrinsic crystallinity of the exfoliated BP on HfO_2_ gate dielectric, which is consistent with previous XPS measurements on BP bulk crystals^28^,^29^. To understand the interaction between Hf and P, first-principles calculations were performed within the density-functional theory (DFT) framework. The results show that Hf adatom prefers the adsorption position above the center of P_6_ hexagonal ring or *H*-site of the phosphorene layer (see inset of Fig. 4), which is consistent with other transition metals reported in previous studies^30^. When this occurs, Hf adatom forms strong covalent bond with phosphorene with a binding energy of −4.17 eV. The stronger P-Hf binding has been found to be consistent with a shorter P-Hf bond and larger deformation of phosphorene layer.

To estimate the effective interface state density *D*_*it*_, the subthreshold swing equation 
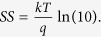
 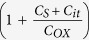 can be employed^31^, where *k* is the Boltzmann constant, *T* is the temperature in Kelvin, *q* is the electronic charge, *C*_*S*_ is the depletion capacitance of BP, *C*_*it*_ is the BP/HfO_2_ interface state capacitance, and *C*_*OX*_ is the unit gate capacitance. When the applied gate voltage approaches the threshold voltage, the thin pristine BP channel is expected to be fully depleted where the depletion capacitance (*C*_*s*_) is small as compared to the interface state capacitance (*C*_*it*_). The effective interface state density *D*_*it*_ at BP/HfO_2_ interface can then be estimated using the expression 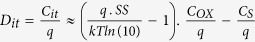. To give better *D*_*it*_ estimation, we have included the contribution of *C*_*s*_ in the calculation where the dielectric constant of BP is taken as 6.1*ε*_*o*_^32^ while the depletion width is equal to the thin BP thickness of 15 nm. Based on the extracted *SS* of 66 mV/dec (annealed) and ~76 mV/dec (un-annealed), the effective interface state density *D*_*it*_ at the BP/HfO_2_ interface is estimated to be ~6.08 × 10^11^ cm^−2^eV^−1^ and ~5.07 × 10^12^ cm^−2^eV^−1^, respectively. The substantial reduction in *D*_*it*_ by ~88% further confirms the effectiveness of Hf adatoms in passivating the P dangling bonds at the BP/HfO_2_ interface, which accounts for the *SS* improvement.

**Figure 5:**
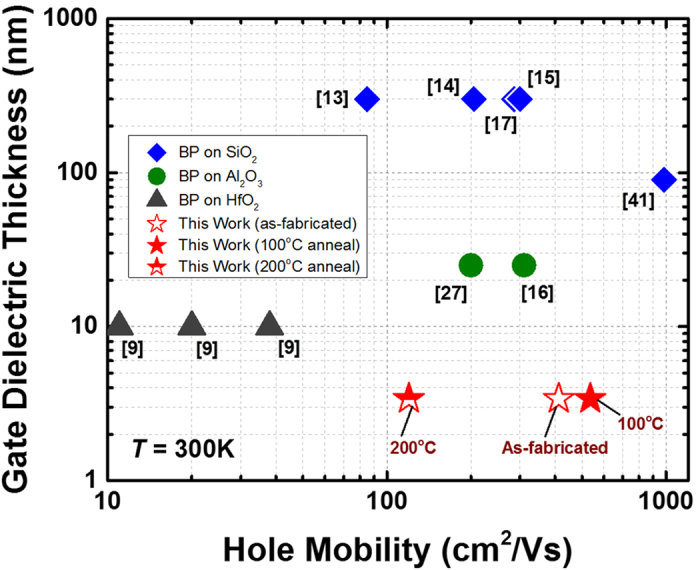
Figure-of-merit showing the relationship between gate dielectric thickness and room temperature hole mobility of black phosphorus transistors. A better BP/HfO_2_ interface quality due to the passivation of phosphorus dangling bonds by hafnium (Hf) adatoms has been shown to result in an enhanced hole mobility over the un-annealed device. A mean mobility of ~413 cm^2^V^−1^S^−1^ and ~536 cm^2^V^−1^S^−1^ were achieved for devices with no anneal and 100 °C anneal, respectively. A further increase of thermal budget beyond 200 °C could significantly degrade the carrier transport due to crystal lattice distortion and impurity scattering effect, leading to a low mean mobility of ~120 cm^2^V^−1^S^−1^.

**Figure 6:**
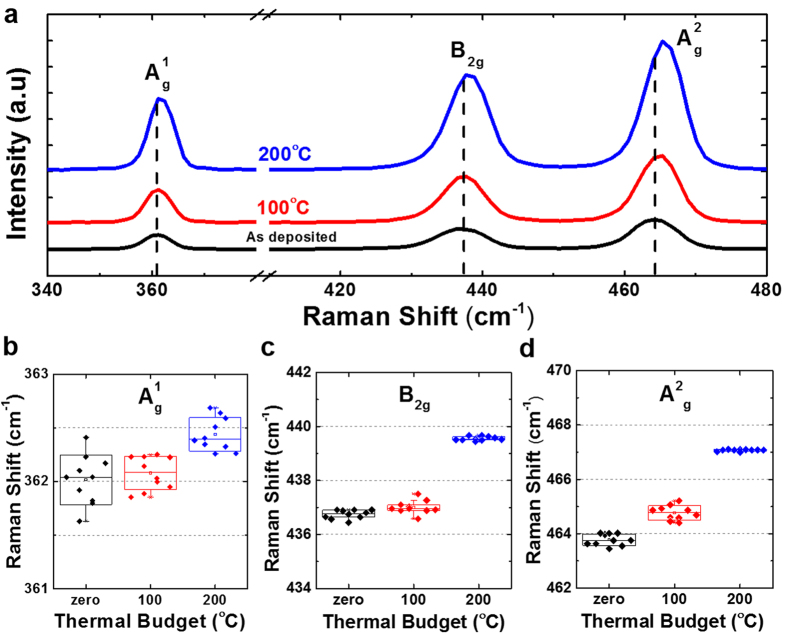
(**a**) Raman spectra of the exfoliated BP nanosheets showed the three characteristic vibrational modes after underwent different thermal anneal for a duration of one minute in nitrogen (N_2_) ambient. The deconvoluted Raman peaks corresponding to the out-of-plane phonon mode A^1^_g_, and in-plane phonon modes B_2g_ and A^2^_g_, are plotted in (**b**–**d**), respectively. A clear Raman peak shift is evident with increasing interface anneal temperature, which indicates film structure modification.

By extracting the slope in the linear region of the transfer curve, the mobility can be evaluated using the expression 
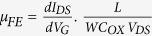
 where *L* is the channel length, *W* is the channel width, *C*_*OX*_ is the capacitance between the channel and the back-gate per unit area and *V*_*DS*_ is the voltage between the source and drain electrodes^5^. The extracted field-effect mobility (μ_FE_) at room temperature is plotted in Fig. 5 and benchmarked against other BP transistors based on traditional SiO_2_ or Al_2_O_3_ gate dielectrics. Even in the absence of thermal treatment, the as-fabricated BP transistor achieves a relatively high *μ*_*FE*_ of 413 cm^2^V^−1^s^−1^. Note worthily, the *μ*_*FE*_ was shown to significantly improve by ~30% to 536 cm^2^V^−1^s^−1^ when the device was treated at an elevated temperature of 100 °C in nitrogen (N_2_) ambient. This is believed to be a record-high hole mobility among other reported data based on HfO_2_ or Al_2_O_3_ high-*k* gate dielectrics at room temperature (*T* = 300 K). Such an improvement was believed to be attributed to a better BP/HfO_2_ interface as a result of phosphorus dangling bonds passivation as discussed in the preceding section. However, a further increase in the thermal budget has been found to degrade the carrier mobility. Noticeably, the *μ*_*FE*_ was drastically reduced to 120 cm^2^V^−1^s^−1^ after going through a 200 °C anneal. In order to understand the underlying mechanisms, the BP film structure properties were analyzed using confocal Raman spectroscopy. Figure 6(a) plots the Raman spectra of the BP flake formed on 3.4 nm HfO_2_ gate dielectric with a 30 nm SiO_2_ passivation layer. When annealed at an elevated temperature, a shift in the Raman phonon peak positions corresponding to A^1^_g_, B_2g_ and A^2^_g_ was clearly evident which is indicative of the film structure modification. A deconvolution of the characteristics out-of-plane mode (A^1^_g_) and in-plane modes (B_2g_ and A^2^_g_) in Fig. 6(a–c), respectively, further confirms the Raman peak position shifts. Interestingly, the samples in this work exhibited a general blue shift with increasing temperatures up to 200 °C. A significant blue shift of 0.53 cm^−1^, 1.0 cm^−1^, and 1.1 cm^−1^ for the A^1^_g_, B_2g_ and A^2^_g_ vibrational modes, respectively, was observed as compared to the un-annealed case. In contrast, when a conventional SiO_2_ dielectric was utilized, both Dattatray^21^ and Zhang *et al*.^22^ experimentally showed a rather linear red shift in all three vibrational modes with increasing temperatures from 77 K up to 673 K.

To understand this shift further, the first-principles linear response method from Fei *et al*.^33^ can be employed. In particular, the effect of zigzag and armchair strain on the puckered honeycomb structure of the black phosphorus will lead to a shift in the Raman peak positions of the characteristics phonon modes depending on the nature of the strain (tensile or compressive). Comparing our results to the first-principles studies, it appears to indicate the presence of compressive strain in both the zigzag and armchair directions for an anneal temperature up to 200 °C, which is clearly evident from the blue shift of the A^1^_g_, B_2g_ and A^2^_g_ modes. This could be attributed to the out-diffusion of hafnium (Hf) adatoms from the underlying high-*k* gate dielectric which compresses the BP channel. According to Clementi *et al*.^34^, the atomic radii of the hafnium (Hf) atoms is 2.08 Å, which is almost two times that of the silicon atoms. In comparison, the atomic radii of the oxygen atoms is 0.48 Å, significantly lower than the hafnium (Hf), silicon (Si), and even the phosphorus (P) atoms (0.98 Å). Hence, in the event of out-diffusion into the BP film, the atom with the largest atomic radii (Hf in our case) is likely to dominate the compressive strain observed. Thus, the incorporation of Hf with a larger atomic radii will be expected to result in crystal lattice distortion in the zigzag direction which will lead to elastic strain effect. However, with a further increase in strain beyond its critical limit, plastic relaxation will eventually occur which causes the crystal lattice to be distorted. When this occurs, the crystalline quality of black phosphorus would be expected to degrade, as evident by the broadening of the full-width at half maximum (FWHM) for both the B_2g_ and A^2^_g_ vibrational modes^35^.

**Figure 7:**
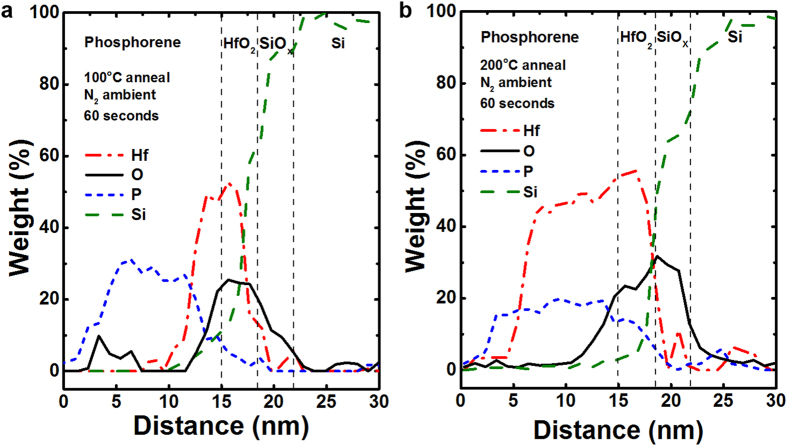
The energy-dispersive X-ray (EDX) spectroscopy measurement confirms the out-diffusion of Hf adatoms from the underlying HfO_2_ gate dielectric into BP film when subjected to thermal treatment. (**a**) With a lower thermal budget of 100 °C, the out-diffused Hf adatoms were predominantly found at the BP/HfO_2_ interface which promotes the passivation of P dangling bonds. (**b**) However with a further increase of thermal budget to 200 °C, a substantial out-diffusion of Hf adatoms was detected in the BP channel which is responsible for the carrier transport degradation in BP transistors.

To confirm the out-diffusion of adatoms, energy-dispersive X-ray spectroscopy (EDX) measurements were performed on the annealed samples to analyze the BP/HfO_2_ interface, as plotted in Fig. 7. It is worthy to note that the degree of hafnium (Hf) and oxygen (O) out-diffusion from the underlying HfO_2_ gate dielectric depends strongly on the thermal budget^38^,^39^. When annealed at a temperature of 100 °C, the out-diffused Hf adatoms were predominantly found at the BP/HfO_2_ interface which could promote the passivation of P dangling bonds [Fig. 7(a)]. However, when the thermal budget was further increased to 200 °C, a substantial amount of Hf adatoms were found to diffuse deeper into the BP channel [Fig. 7(b)]. Given the puckered honeycomb structure of BP, these out-diffused adatoms are likely to preferentially occupy the *H*-site in the zigzag (B_2g_ and A^2^_g_) direction which has been theoretically predicted for most transition metals in phosphorene^30^. This is believed to be responsible for the compressive strain observed in the BP nanosheet after a high temperature anneal. Moreover, these out-diffused adatoms could also act as impurities scattering centers which would detrimentally increase the channel resistance and degrade the carrier transport properties.

**Figure 8:**
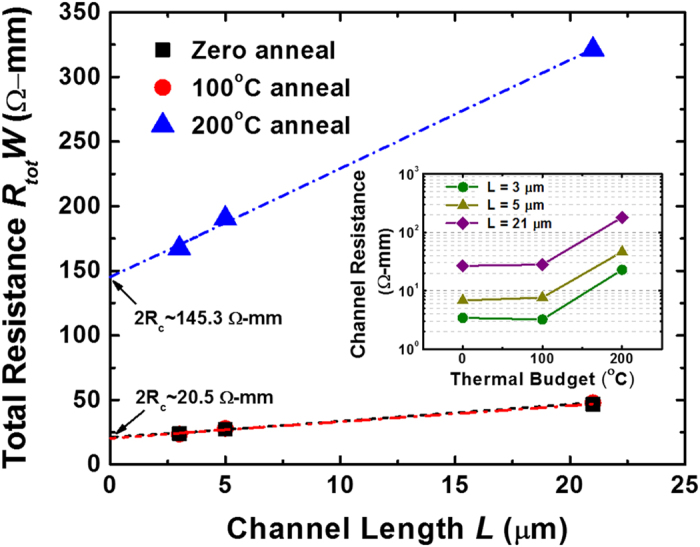
Using the transfer length method (TLM), the total normalized resistance (*R*_*tot*_*W*) was plotted as a function of channel length (*L*). Comparable contact and channel resistance were achieved for a thermal anneal of 100 °C as compared to the un-annealed case. However, an elevated thermal treatment at 200 °C could result in a detrimental change to the BP film structure, which leads to both channel and contact resistance degradation.

To gain a further insight into the impact of impurities scattering centers on channel resistance, the transfer length method (TLM) was employed in which the total normalized resistance (*R*_*tot*_*W*) was plotted as a function of channel length (*L*) as shown in Fig. 8. Using this approach, the contact resistance can be determined by the *y*-intercept of the width normalized total resistance. The channel resistance can also be determined by subtracting the contact resistance from the total resistance, which is summarized in the inset of Fig. 8 as a function of thermal budget. When subjected to a thermal treatment at 100 °C, the BP structural integrity was found to remain intact, as evident by the comparable channel and contact resistances over the un-annealed case. On the contrary, a high degree of crystal lattice distortion was present in the BP film after a 200 °C thermal treatment, as supported by the Raman phonon shifts in Fig. 6. This is found to be detrimental for device performance, which is evident from the higher channel resistance by 5× as compared to the un-annealed devices. Apart from channel resistance degradation, the contact resistance has also been found to increase after a 200 °C anneal in which the 2R_c_ approaches ~145.3 Ω-mm. This is substantially higher than the contact resistance measured in devices which underwent 100 °C or no anneal, where the 2R_c_ was merely ~20.5 Ω-mm. Such a degradation is likely to be attributed to the crystal lattice distortion which causes a poorer interface quality between the metal and the BP layer. The adsorption of Hf adatoms in BP could also additionally cause an increased impurities scattering effect^37^ which would compromise the carrier transport in BP transistors. This is consistent with the lower hole mobility of 120 cm^2^V^−1^s^−1^ and poorer subthreshold swing *SS* of ~780 mV/dec discussed earlier. Hence, an optimization of the thermal treatment process is critically important to effectively engineer the interface quality between BP and HfO_2_ high-*k* gate dielectric for enabling further performance boost.

## Conclusions

In conclusion, we have experimentally demonstrated black phosphorus transistor with enhanced carrier transport through interface engineering between BP and high-*k* gate dielectric. A high room temperature hole mobility of ~536 cm^2^V^−1^s^−1^ and a near ideal subthreshold swing of ~66 mV/dec were simultaneously achieved. Such a performance enhancement is attributed to the better interface quality between BP and HfO_2_ as a result of phosphorus dangling bonds passivation by hafnium adatoms, which leads to a significant reduction in interface state density. Excessively high treatment temperature could promote the out-diffusion of hafnium adatoms into the BP channel, which distorts its crystal lattice and results in carrier transport degradation. Our study contributes to the development of BP-based transistor with enhanced performance through interface engineering.

## Experimental Methods

### Sample Preparation and Device Fabrication

Black phosphorus flake with a thickness of ~15 nm was micromechanically exfoliated onto p-type silicon substrate with an ultra-thin HfO_2_ high-*k* gate dielectric of ~3.4 nm. The HfO_2_ was deposited by atomic layer deposition (ALD) process. Utilizing tetrakisethylmethylamino hafnium (TEMAH) and water precursors at a deposition temperature of 250 °C, a deposition rate of ~1 Å/cycle is achieved. For every cycle of deposition, the TEMAH precursor and the water precursors are pulsed at 0.015 seconds and 0.01 seconds respectively, followed by a waiting time of 10 seconds. During the deposition, nitrogen with a flow rate of 20 sccm is utilised as the carrier gas. This was followed by a sequential cleaning in acetone followed by isopropyl alcohol before the deposition of metal electrodes using nickel (Ni) through a combination of electron beam lithography (JEOL, JBX-6300FS) and electron-beam evaporation (Oerlikon, Univex 450B). The remaining photoresist was then lifted-off to complete the device fabrication. A 30 nm silicon-dioxide passivation layer was then deposited at room temperature using electron-beam evaporation (Oerlikon, Univex 450B) over the entire BP transistor to protect the device from photo-oxidation in ambient condition^23^. Subsequently, the devices were subjected to interface thermal treatment. The anneal conditions were varied from zero to 200 °C for one minute in nitrogen (N_2_) ambient to investigate the impact of thermal treatment on the structural and electrical properties of the BP transistors. Confocal Raman spectroscopy (WITec, Alpha300R) was utilized to analyze the influence of interface anneal temperature on the Raman shifts corresponding to the out-of-plane (A^1^_g_), and in-plane (B_2g_, A^2^_g_) phonon modes^36^.

## Additional Information

**How to cite this article**: Ling, Z.-P. *et al*. Interface Engineering for the Enhancement of Carrier Transport in Black Phosphorus Transistor with Ultra-Thin High-*k* Gate Dielectric. *Sci. Rep.* **6**, 26609; doi: 10.1038/srep26609 (2016).
